# Interleukin-6 signaling regulates hematopoietic stem cell emergence

**DOI:** 10.1038/s12276-019-0320-5

**Published:** 2019-10-24

**Authors:** Ruxiu Tie, Honghu Li, Shuyang Cai, Zuyu Liang, Wei Shan, Binsheng Wang, Yamin Tan, Weiyan Zheng, He Huang

**Affiliations:** 1 Bone Marrow Transplantation Center, The First Affiliated Hospital, Zhejiang University School of Medicine, No. 79 Qingchun Road, 310003 Hangzhou, China; 2 Institute of Hematology, Zhejiang University, No. 866 Yuhangtang Road, Hangzhou, 310058 China; 3 Zhejiang Engineering Laboratory for Stem Cell and Immunity Therapy, No. 866 Yuhangtang Road, Hangzhou, 310058 China; 4 Stem Cell Institute, Zhejiang University, No. 866 Yuhangtang Road, Hangzhou, 310058 China; 5 Department of Hematology, the Second Clinical Medical College, Shanxi Medical University, No. 382 Wuyi Road, 030000 Taiyuan, China

**Keywords:** Haematopoietic stem cells, Zebrafish

## Abstract

Hematopoietic stem cells (HSCs) produce all lineages of mature blood cells for the lifetime of an organism. In vertebrates, HSCs derive from the transition of the hemogenic endothelium (HE) in the floor of the embryonic dorsal aorta. Most recently, a series of proinflammatory factors, such as tumor necrosis factor-α, interferon-γ, and Toll-like receptor 4, have been confirmed to play a key role in HSC specification. However, the full complement of necessary signaling inputs remains unknown to date. Here, we show that interleukin-6R (IL6R) via IL6 is required and sufficient for HSC generation. We found that Notch activates IL6R by regulating its expression in the HE and in HSCs. The secretion of IL6 mainly originates from HSC-independent myeloid cells, but not from HSCs and their adjacent vascular endothelial cells. In addition, blocking IL6 signaling does not affect vascular development or the production of primitive erythrocytes. Taken together, our results uncover a previously obscure relationship between IL6 signaling and HSC production and provide new insights into HSC regeneration using proinflammatory factors in vitro.

## Blood stem cells: Finding a critical step in development

A molecule that triggers inflammation, interleukin-6 (IL6), is crucial for development of blood stem cells, known as hematopoetic stem cells (HSCs). HSCs can differentiate into any type of blood or immune cell, and have enormous therapeutic potential. Although some of the signaling molecules that trigger their development are known, HSCs have yet to be grown under laboratory conditions, so knowledge gaps remain. Based on clues that IL6 might be one of the missing signals, He Huang at the Zhejiang University School of Medicine, Hangzhou, China, and coworkers investigated how IL6 affects HSC development in zebrafish, a common model of development. Zebrafish embryos lacking IL6 were deficient in HSCs, but showed normal blood vessel growth. Treating with IL6 restored HSC production. These results represent another step toward being able to grow HSCs for transplantation and therapy.

## Introduction

Adult hematopoietic stem cells (HSCs) have the ability to self-renew and differentiate into all blood and immune cells throughout life. In vertebrate embryos, naive HSCs arise de novo from the hemogenic endothelium (HE) in the ventral wall of the dorsal aorta^1–4^. Thus far, many signaling pathways have been implicated in this complex process. However, a more comprehensive understanding remains needed, partly because of the failure to reproduce healthy HSCs for HSC transplantation (HSCT) in vitro^5–8^.

Under stress-induced hematopoiesis, adult short-term HSCs and multipotent progenitor cells (MPPs) can directly sense bacterial and viral components and systematically elevate cytokine expression through Toll-like receptors (TLRs)^9–11^. Numerous cytokines, such as interleukin-6 (IL6), tumor necrosis factor-α (TNF-α), interferon-γ (IFN-γ), granulocyte–macrophage colony-stimulating factor (GM-CSF), and IFN-α, have been identified to regulate the proliferation and differentiation of adult hematopoietic stem and progenitor cells (HSPCs) in a paracrine manner^11^. These studies prompted us to investigate whether these cytokines play a role in HSC production under physiological conditions. Most recently, several studies have demonstrated that proinflammatory signaling factors, including granulocyte-CSF(G-CSF), TNF-α, IFN-γ, and TLR4, can positively specify embryonic HSC fate^12–16^. Importantly, all of these proinflammatory factors are functionally associated with Notch signaling.

IL6 is a prominent proinflammatory cytokine that plays a pivotal role in controlling immunity and inflammation^17,18^. IL6 exerts its functions via the engagement of the type 1 cytokine a-receptor subunit (IL6R) and the signal-transduction β-receptor subunit (GP130)^19–22^. IL6, IL6R, and GP130 (Il6, Il6r, and Gp130 utilizing zebrafish nomenclature) are well conserved in all vertebrate organisms. The targeted disruption of GP130 leads to embryonic lethality, presenting hypoplastic ventricular myocardium and reduced HSPCs in the liver, whereas more detailed information is absent^23^. It is well known that GP130 is a common signal transducer for IL6 family of cytokines, including IL6, IL11, IL27, leukemia inhibitory factor, oncostatin M, ciliary neurotrophic factor, cardiotrophin 1, and cardiotrophin-like cytokine. Thus, we fail to discern which signaling pathway may participate in embryonic hematopoiesis. One report demonstrates that adult HSPCs from Il6-deficient mice have defective capacities in proliferation and self-renewal compared with those of wild-type controls^24^. However, it is still unknown whether IL6 signaling affects embryonic HSC emergence. With the diverse combinations with other classical factors, IL6 has been used to promote the proliferation of HSPCs in vitro^25–27^. Most recently, another study demonstrated that platelet-derived growth factor receptor-β signaling promotes embryonic HSC production by regulating *il6* expression^28^, but this study did not depict the role of Il6 signaling in HSC emergence. All of the evidence above suggests that Il6 signaling may participate in embryonic HSC specification and maintenance, and a comprehensive blueprint needs to be explored.

During embryonic development, hematopoiesis occurs in at least two waves, known as primitive and definitive hematopoiesis, which are conserved in all vertebrates. Primitive hematopoiesis produces mainly primitive erythrocytes, macrophages (MFs) and megakaryocytes (Mks)^29^. Definitive erythromyeloid progenitors (EMPs)^30^ emerge distinct from both primitive hematopoiesis and HSCs, which produces mainly definitive erythrocytes, MFs, Mks, and neutrophils^29,31^. Zebrafish have emerged as a model for the study of hematopoietic disorders and embryonic hematopoiesis^32–35^. Here, we use the model to present a previously unappreciated requirement for Il6 signaling in HSC specification and emergence. We found that Notch activates *Il6r* by regulating its expression in the HE and in HSCs, and Il6 is mainly secreted by neutrophils and MFs from primitive and definitive EMP hematopoiesis. Altogether, our study reveals an undiscovered function of Il6 signaling in the birth of HSCs under nonpathogenic conditions.

## Materials and methods

### Zebrafish husbandry and strains

Zebrafish strains were raised as described^36^, and the adult fish were maintained in accordance with the relevant guidelines of the Laboratory Animal Center of Zhejiang University. The study was approved by the Institutional Animal Care and Use Committee of the Laboratory Animal Center, Zhejiang University (Hangzhou, China). See the Supplemental Information for a description of the transgenic lines used in this study (Table S1).

### Morpholino and mRNA injections

Antisense morpholinos (MOs) used in our study were purchased from Gene Tools, including Standard Control MO (Gene Tools), *irf8* MO^37^, *pu.1* MO^38^, *tnfα* MO^13^, *notch1a* MO^13^, *il6* MO, and *il6r* MO (this work). The MOs were diluted in diethyl pyrocarbonate-treated water at a concentration of 0.2 mM (Standard-MO), 0.5 mM (*tnfα* MO), 1.2 mM (*notch1a* MO), 0.6 mM (*il6* MO), 0.4 mM (*il6r* MO), 1.4 mM (*irf8* MO), and 2 mM (*pu.1* MO) with phenol red solution, and 1 nl was injected into the yolk ball of one- or two-cell-stage embryos. The MO sequences are listed in Table S2. The *iI6r* MO validation was performed using reverse transcription-polymerase chain reaction (Table S3).

For messenger RNA (mRNA) generation, the total RNA of embryos at 28 hr postfertilization (hpf) was isolated from MO-injected embryos and reverse transcribed into complementary DNA (cDNA). Two primers (Table S4) were used to amplify the *il6r* opening reading frame. The PCR product was cloned into the pCS2 + vector and confirmed by bidirectional sequencing (Vazyme). The pCS2^+^-*il6r* vector was linearized by *Not*I and purified (Takara Purification Kit). Capped *il6r* mRNA was produced using the SP6 mMESSAGE mMACHINE Kit (Life Technologies), and then 200 pg purified *il6r* mRNA was injected into one-cell stage embryos at the yolk/cytoplasm boundary for rescue experiments.

### Whole-mount RNA in situ hybridization

Probes for the *runx1*, *cmyb*, *kdrl*, *efnb2a*, *dlc*, *gata1a*, *mpx*, *l-plastin*, *rag1*, and *foxn1* transcripts were generated using a DIG RNA Labeling Kit (Roche Applied Science) from linearized plasmids. Whole-mount in situ hybridization (WISH) was carried out as described previously^39^. The embryos were observed using a Leica M165C stereomicroscope and imaged with a DFC295 color digital camera (Leica). Qualitative phenotypes for individual embryos (*n* ≥ 10 embryos/condition, *n* ≥ 3 replicate clutches unless otherwise indicated) were assessed as relatively high (up)/medium (normal)/low (down)/absent in expression compared with control siblings at noted stages and were depicted graphically as the percentage falling into each of three phenotypic expression bins; “medium” expression was the most representative qualitative phenotype in the normal bell curve distribution of each control group per experiment.

### Enumeration of HSCs

Confocal microscopy was performed on *cmyb:GFP; kdrl:mCherry* double-transgenic animals^1^, *tp1:eGFP; kdrl:mCherry* double-transgenic animals, and *mpeg1:eGFP* transgenic animals. Z sections of the DA region or whole embryos were captured on a Leica SP5 microscope (Leica) using Volocity Acquisition, Visualization, and Restoration software (Improvision) and were manually counted.

### Fluorescent visualization of HSPCs, neutrophils, and T cells

To visualize HSPCs, neutrophils, and T cells, we used these transgenic models, including *cd41:eGFP*; *lyz:dsred* embryos at 72 hpf and *lck:GFP* larvae at 5 days *postfertilization* (dpf), respectively, were anesthetized in tricaine (200 μg/ml) and observed using a Leica MZ16FA stereomicroscope.

### FACS and quantitative real-time PCR

Fluorescence-activated cell sorting (FACS) analysis was performed using *fli1a:eGFP*, *mpx:eGFP*, and *mpeg1:eGFP* transgenic embryos. First, ~50–100 embryos were stored on ice in 500 μl phosphate-buffered saline (PBS) containing 2% fetal bovine serum and dissociated using a P1000 pipette. The resulting suspension was filtered with a 40 μm cell strainer and resuspended in 1× PBS. Then, we isolated target cells by using a FACSAria cell sorter (BD Biosciences, San Jose, CA). Subsequently, we used an RNeasy Mini Kit (Qiagen) to extract mRNA. During the process, 500 ng polyinosinic acid potassium salt (Sigma) was added to the RLT buffer for each sample. cDNA was synthesized using a Quantitect cDNA Synthesis Kit (Qiagen) and diluted five times as the template. Quantitative PCR (qPCR) was performed using the Bio-Rad CFX96 real-time PCR system. Relative expression was calculated by the 2^−△△C(T)^ method, and *ef1a* was used as the housekeeping gene^13^. The PCR primers used in this study are listed in supplemental Table S5.

### Detection of apoptotic cell death by TUNEL labeling

TUNEL assays were performed as described previously^40^. The *fli1a:eGFP* transgenic embryos were fixed in 4% paraformaldehyde (PFA) at 4 °C overnight and then dehydrated in 100% methanol at −20 °C for at least 2 h. After gradual rehydration, the embryos were washed three times with PBST and treated with proteinase K (10 μg/ml) for 10 min. At room temperature, the permeabilized embryos were refixed in 4% PFA for 20 min. After washing 3–6 times with PBST, the embryos were incubated with a mixture containing 45 μl labeling solution and 5 μl enzyme solution (In Situ Cell Death Detection Kit TMR Red; Roche) at 4 °C overnight. Finally, after washing three times with PBST, the stained embryos were imaged by confocal microscopy.

### Chemical treatment

Experimental embryos were exposed to the Notch-specific inhibitor DAPT (*N*-[*N*-(3,5-difluorophenacetyl)-lalanyl]-*S*-phenylglycine *t*-butyl ester) at 100 μM (dissolved in dimethyl sulfoxide (DMSO), Sigma) from the 10-somite stage to 48 or 72 hpf in multiwell plates of Danieau’s solution. Siblings treated with 1% DMSO in Danieau’s solution were regarded as controls.

### Statistical analyses

Statistical analysis was performed using the GraphPad Prism software (GraphPad). Two-tailed Student’s *t* tests were used for pairwise comparisons. In all statistical figures, solid red bars represent the mean, and error bars denote the SEM. The data are presented as the means ± SEM. *P < 0.05 was considered statistically significant, **p < 0.01, ***p < 0.001, ****p < 0.0001; n.s., not significant.

## Results

### Il6 signaling through il6r is required for HSC production

Most recently, several studies in mouse and zebrafish embryos demonstrated that proinflammatory factors are required for HSC emergence^12–14,16^. IL6 is implicated in adult HSC proliferation and differentiation, but its detailed function in embryonic HSC development remains unclear. One recent published report isolated zebrafish *kdrl*^+^ *cmyb*^−^ vascular endothelial cells (ECs) and *kdrl*^+^ *cmyb*^+^ HSCs from double-transgenic *kdrl:mCherry* and *cmyb:eGFP* embryos at 36 hpf by FACS and performed qPCR for *il6*, *il6r*, and *gp130*. The transcript *il6r* was enriched in HSCs compared to ECs, while the expression differences in the other two transcripts were not observed between the two groups^28^. To further investigate the expression of these genes, we analyzed published single-cell transcriptomic data on mouse embryonic HSCs at different stages^41^. Compared to vascular ECs, the expression of *il6r* and *gp130* was significantly higher in developing HSCs, whereas *il6* was too low to be detected in HSCs at all time points (Fig. S1). These results suggest that Il6 may play an important role in embryonic HSC emergence in a paracrine way.

**Fig. 1:**
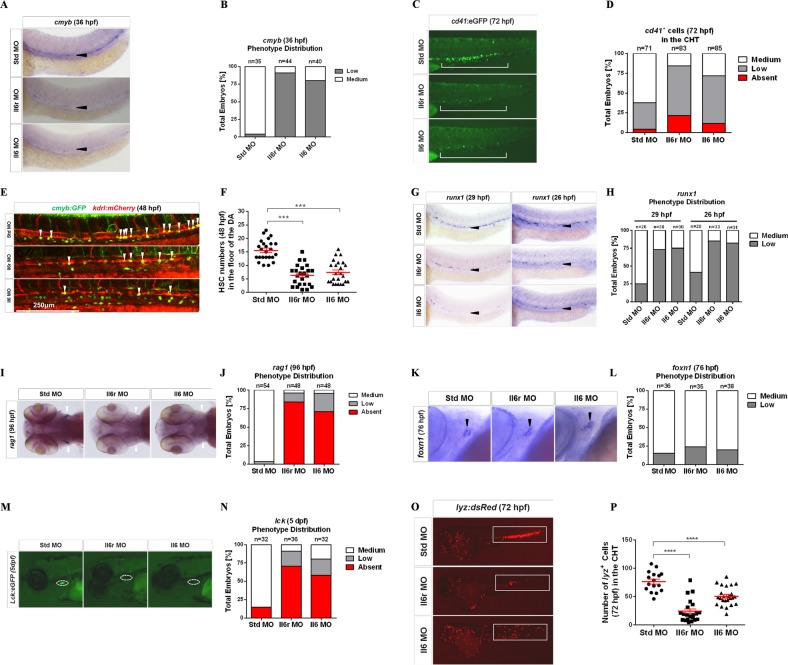
Il6r and Il6 are required for HSC generation. **a** Standard control (Std), Il6r, and Il6 morphants were detected by WISH for *cmyb* expression in the dorsal aorta (DA) at 36 hpf. Black arrowheads denote *cmyb*^+^ HSCs. **b** Qualitative phenotype distribution of the embryos (*n* above the bar graph denotes the number of embryos per group) from **a** scored with medium (normal) and low (down) *cmyb* expression. Medium, white bar; low, gray bar. **c** *cd41:eGFP* transgenic embryos were injected with Std, Il6r, and Il6 MOs and visualized at 72 hpf in caudal hematopoietic tissue (CHT). The white long line denotes *cd41*^+^ cells in the CHT region. **d** Qualitative phenotype distribution of embryos from **c** scored as in **b**. Medium, white bar; low, gray bar; absent, red bar. **e** Confocal tracking of HSC numbers in the floor of the DA region from individual *cmyb:GFP; kdrl:mCherry* double-transgenic embryos at 48 hpf. White arrowheads denote *cmyb*^*+*^*, kdrl*^*+*^ HSCs, scale bar represent 250 μm. **f** Enumeration of *cmyb*^*+*^*, kdrl*^*+*^ HSCs from **e**, bars represent the means ± SEM of Std (*n* = 24), Il6r (*n* = 24), and Il6 (*n* = 24) morphants. ***P < 0.001. **g** Std, Il6r, and Il6 morphants were examined by WISH for *runx1* expression at 29 and 26 hpf, respectively. Black arrowheads represent HSCs in the floor of the DA region. **h** Qualitative phenotype distribution of embryos from **g** scored as in **d**. **i**, **k** Representative WISH images for *rag1*^+^ T lymphocyte (**i**) (white arrowheads) and *foxn1*^+^ thymic epithelial markers (black arrowheads) at 96 and 76 hpf, respectively. **j**, **l** Qualitative phenotype distribution of embryos from **i**, **k** scored as in **d**. **m** Representative images of *lck*^+^ T lymphocytes in Il6r and Il6 morphants compared to Std controls at 5 dpf. White dashed lines denote the thymus. **n** Qualitative phenotype distribution of embryos from **m** scored as in **d**. **o** Representative images of *lyz:dsRed* transgenic embryos injected with Std, Il6r, and Il6 MOs. White blocks denote the CHT and trunk regions. **p** Enumeration of *lyz*^*+*^ myeloid cells shown in **o**. Bars represent the means ± SEM of Std (*n* = 16), Il6r (*n* = 21), and Il6 (*n* = 24) morphants. ****P < 0.0001

To investigate whether Il6 signaling was required for HSC specification, we performed loss-of-function experiments for Il6 and its receptor Il6r using targeted MOs. In the zebrafish embryo, HSCs are marked by the expression of *cmyb* along axial vessels^42^. Using WISH, we found that the expression of *cmyb* in or near the floor of the DA region at 36 hpf was dramatically reduced in Il6- and Il6r-deficient embryos compared with their wild-type siblings (Fig. 1a, b), indicating that the action of Il6 through Il6r is required in HSC development. A similar result was observed by the quantitation of *cd41:eGFP*^+^ HSPCs in the caudal hematopoietic tissue (CHT) region at 72 hpf^43^, as determined by fluorescence microscopy (Fig. 1c, d). The specificity of *il6r* MO was confirmed by splice product detection (Fig. S1B).

To further verify the reduction of HSCs in Il6- and Il6r-deficient embryos, we directly observed emerging HSCs from the floor of the DA region in *kdrl:mCherry; cmyb:GFP* double-transgenic embryos at 48 hpf by confocal microscopy^1^. Consistent with the above results, the number of *kdrl; cmyb* double-positive HSCs was significantly less than those in control embryos (Fig. 1e, f). This reduction might be due to a defect in the initial HSC specification. To address this question, we performed WISH to detect the expression of *runx1*, which is a nascent HSC marker at an earlier developmental stage^1,2,4,44^. The expression of *runx1* was significantly reduced in the aorta floor at 26 and 29 hpf (Fig. 1g, h), indicating that Il6 signaling is required during the earliest stages of HSC specification.

We next investigated subsequent larval stages by detecting the expression of *rag1* and *lck*^45^, two markers located in developing thymocytes because the T cell lineage derives exclusively from HSCs^43,46^. *Rag1* expression was completely or nearly absent in Il6- and Il6r-deficient animals at 96 hpf (Fig. 1i, j); however, the expression of the thymic epithelial cell marker *foxn1* was normal in all morphants (Fig. 1k, l). The results were further confirmed by tracking T cell lineage development in *lck:eGFP* transgenic embryos at 5 dpf (Fig. 1m, n).^45^ In addition, we assessed myeloid lineage cell development using *lyz:dsRed* transgenic embryos in CHT, and the number of neutrophils was significantly reduced in Il6- and Il6r-deficient animals at 72 hpf (Fig. 1o, p). Together, these results demonstrate that Il6 signaling via Il6r is essential for both the specification and subsequent maintenance of HSCs in the embryonic development stage.

### Il6 signaling through Il6r is required for HSC-independent neutrophils but not MFs and primitive erythrocytes

**Fig. 2:**
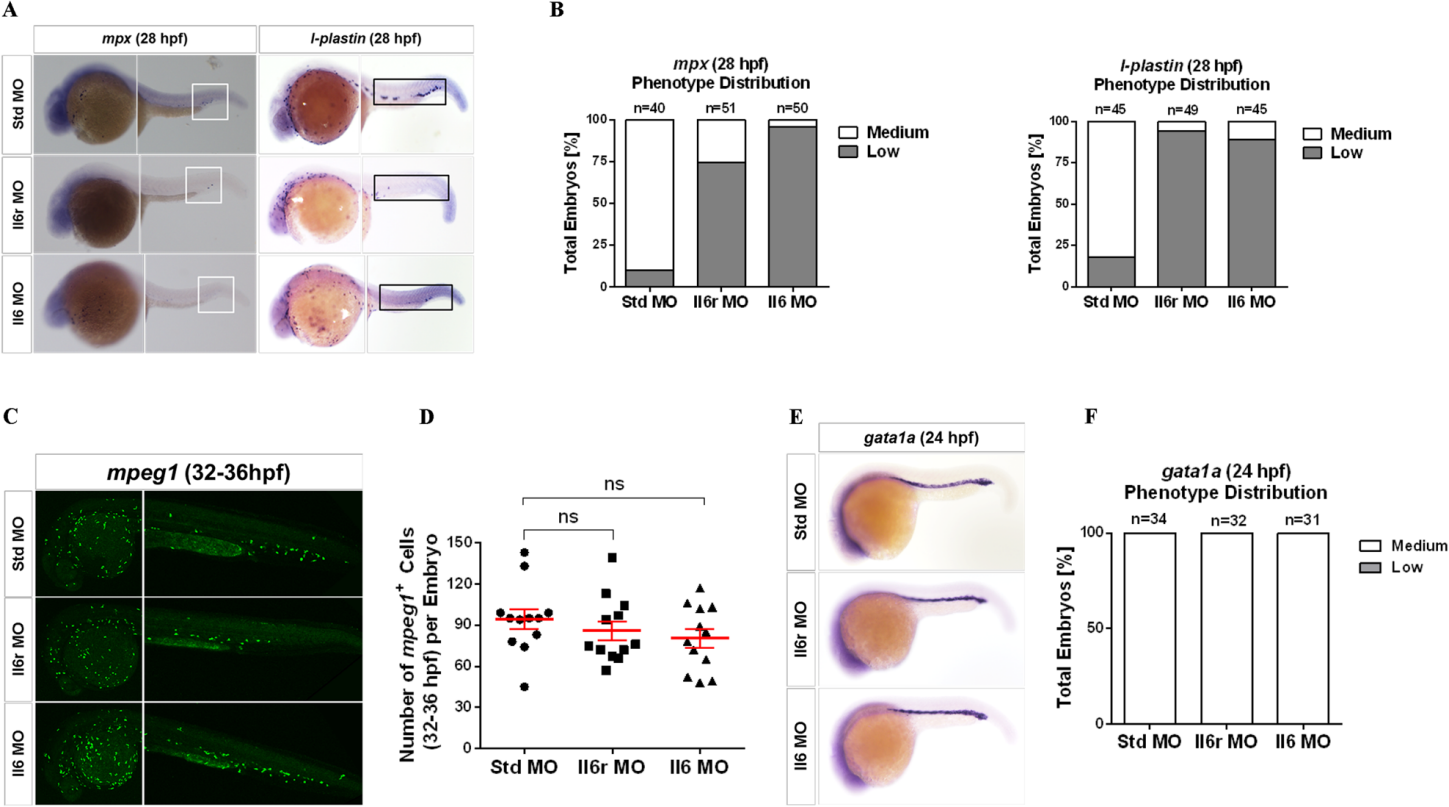
Il6 signaling is required for HSC-independent neutrophil development, but is not indispensable for the development of HSC-independent macrophages and primitive erythrocytes. **a** Representative images of Std, Il6r, and Il6 morphants detected by WISH for *mpx* and *l-plastin* expression at 28 hpf. The white block denotes the posterior blood island (PBI) region, and the black block denotes the PBI and trunk regions. **b** Qualitative phenotype distribution of embryos from **a** scored with medium (normal) and low (down) *mpx and l-plastin* expression, respectively. Medium, white bar; low, gray bar. **c** Confocal tracking of HSC-independent macrophages in *mpeg1*:*GFP* transgenic embryos injected with Std, Il6r, and Il6 MOs at 32–36 hpf. **d** Enumeration of *mpeg1*^*+*^ macrophages cells in the yolk sac, PBI, and trunk regions from **c**. Bars represent the means ± SEM of Std (*n* = 12), Il6r (*n* = 12), and Il6 (*n* = 12) morphants. n.s., Not significant. **e** Representative images of Std, Il6r, and Il6 morphants interrogated by WISH for *gata1a* expression at 24 hpf. **f** Qualitative phenotype distribution of embryos from **e** scored as in **b**

To further dissect the role of Il6 signaling in embryonic hematopoiesis, we evaluated whether Il6 and its receptor Il6r were required for the first wave of hematopoiesis, also named the “primitive wave” due to the lack of upstream multipotent progenitors. The expression of *l-plastin*, a specific marker of myeloid cell lineage, was not significantly affected in the yolk sac of Il6 and Il6r morphants at 28 hpf (Fig. 2a, b). Similar results were observed in terms of primitive neutrophils and MFs using the specific markers *mpx* and *mpeg1*, respectively (Fig. 2a, c, d). Moreover, primitive erythropoiesis was unchanged in all morphants, as assayed by the expression of the erythrocyte-specific marker *gata1a* at 24 hpf (Fig. 2e, f).

In zebrafish embryos, secondary wave hematopoiesis occurs in the posterior blood island (PBI) produced autonomously at ~30 hpf, namely, definitive EMPs, which are distinct from both primitive hematopoiesis and definitive HSCs^47^. We found that the expression of *l-plastin* and *mpx* was decreased significantly in the PBI and trunk regions of Il6- and Il6r-deficient embryos, but the same trend was not observed in *mpeg1*-positive transgenic embryos (Fig. 2a–d), suggesting that the development of HSC-independent neutrophils from EMPs is also dependent on Il6 signaling. Overall, these results indicate that Il6 signaling is dispensable for primitive hematopoiesis and indispensable for definitive EMPs in the zebrafish embryo.

### Il6r- and Il6-deficient embryos display normal vasculogenesis

**Fig. 3:**
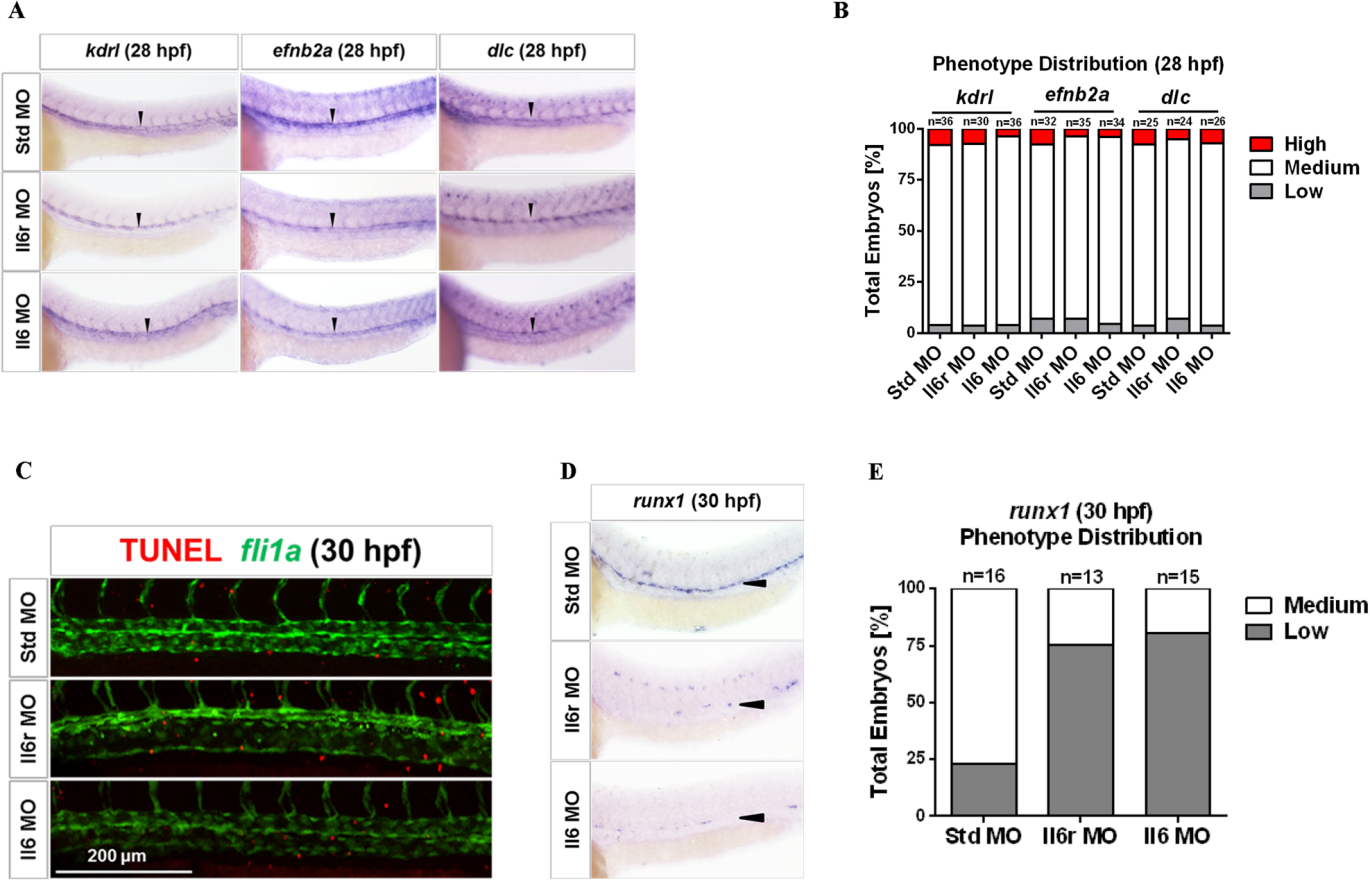
Signaling through Il6r regulates HSC development under the normal vasculogenesis. **a** Std, Il6r, and Il6 morphants were interrogated by WISH for *kdrl*, *efnb2a*, and *dlc* at 28 hpf. Black arrowheads denote the vascular lines. **b** Qualitative phenotype distribution of embryos from **a** scored with medium, high (up), and low (down) expression of these markers. Medium, white bar; high, red bar; low, gray bar. **c** Maximum projections of the DA region of *fli1a*:eGFP embryos at 30 hpf injected with Std, Il6r, and Il6 MOs and assayed for immunohistochemistry for GFP (green) and TUNEL (red). Scale bar represents 200 μm. **d** Embryos from the same experiment as in **c** were subjected to WISH for *runx1* expression at 30 hpf. Black arrowheads denote HSCs in the floor of the DA region. **e** Qualitative phenotype distribution of embryos from **d** scored as in **b**

HSCs originate in aorta vessels, and many genes affecting vascular or arterial specification also control HSC development^40,47^. No remarkable vascular abnormalities were found in Il6 and Il6r morphants at 28 hpf when assayed by WISH for the vascular endothelial specific marker *kdrl* in this study (Fig. 3a, b). To further determine whether the reduction in HSCs resulted from impaired arterial specification, we used WISH to detect the expression of the arterial-specific markers *efnb2a* and *dlc* at 28 hpf^48^. No obvious alterations were observed among the different groups (Fig. 3a, b). Taken together, these results indicate that Il6 signaling promotes HSC emergence under normal vasculature conditions.

To determine whether the loss of HSCs in the absence of Il6 signaling could be attributed to the apoptosis of ECs, we performed a TUNEL assay using *fli1a:GFP* embryos injected with *il6r* and *il6* MOs. Using confocal microscopy, we found that Il6 and Il6r morphants at 30 hpf did not have increased apoptotic ECs within the DA region (Fig. 3c)^49^. Additionally, we performed WISH for *runx1* in this experiment to confirm the reduction in HSCs in these embryos (Fig. 3d, e). Taken together, these results indicate that the defects of HSC specification in Il6- and Il6r-deficient embryos are not caused by apoptosis of ECs within the vasculature.

### Il6r acts downstream of Notch signaling for HSC production

**Fig. 4:**
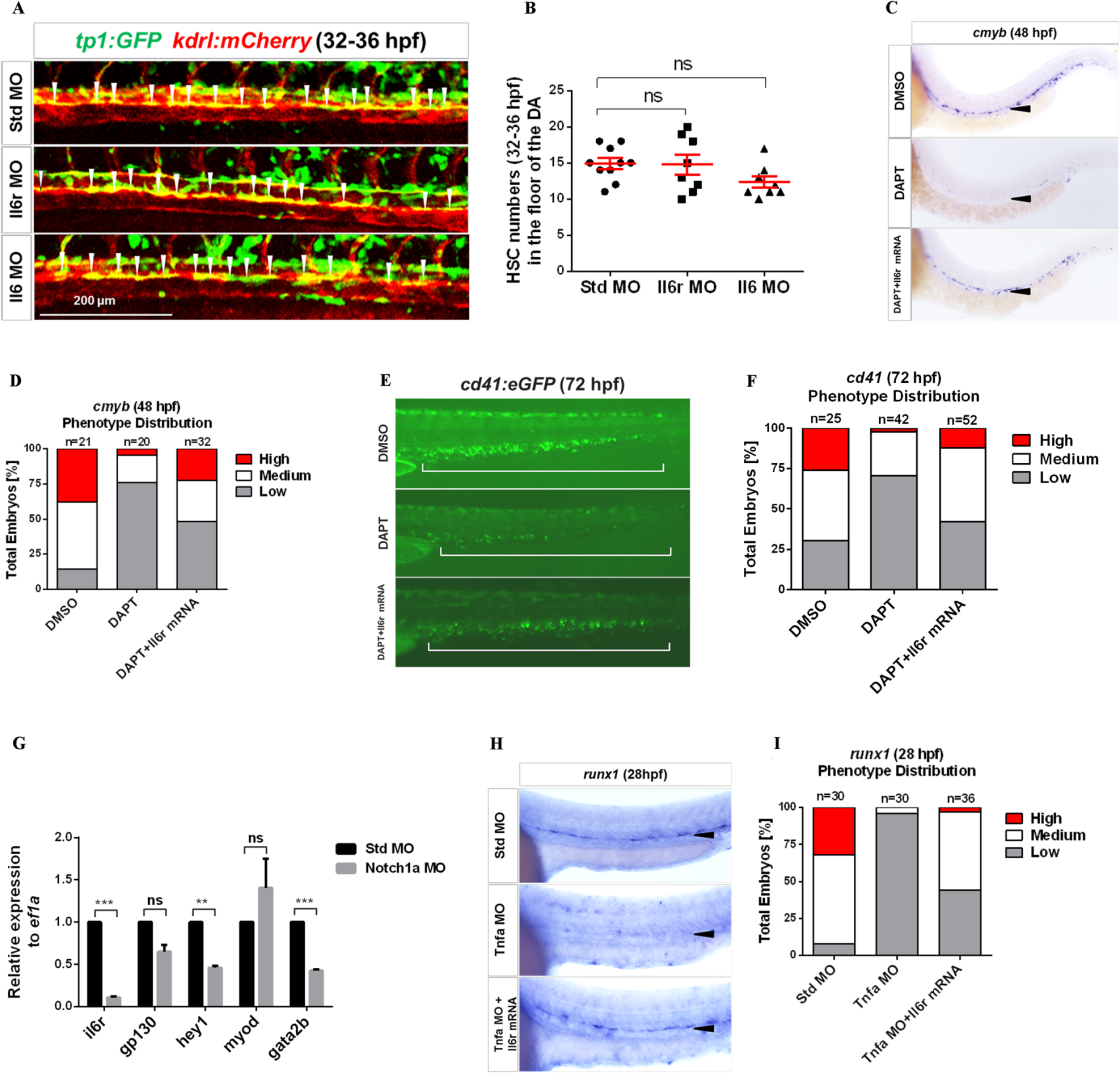
Il6r acts downstream of Notch signaling in HSC development. **a** Confocal tracking of HSC numbers from individual *tp1:eGFP*; *kdrl:mCherry* double-transgenic embryos injected with Std, Il6r, and Il6 MOs at 32–36 hpf. White arrowheads denote *tp1*^+^, *kdrl*^+^ HSCs with active Notch signaling in the floor of the DA region. **b** Enumeration of HSCs from **a**, bars represent the means ± SEM of Std (*n* = 10), Il6r (*n* = 8), and Il6 (*n* = 8) morphants. n.s., Not significant. **c** WISH for *cmyb* in the DA region of DMSO-treated, DAPT-treated, DAPT-treated plus *il6r* mRNA-injected embryos at 48 hpf. Black arrowheads denote HSCs in the floor of the DA region. **d** Qualitative phenotype distribution of embryos from **c** scored with medium, high, and low *cmyb* expression. Medium, white bar; high, red bar; low, gray bar. **e** Representative images of *cd41:eGFP* transgenic embryos treated with DMSO, DAPT, and DAPT plus *il6r* mRNA. Hematopoietic cells in the CHT region were visualized at 72 hpf. The white long line denotes the CHT region. **f** Qualitative phenotype distribution of the embryos from **e** scored as in **d**. **g** The expression of genes including *il6r*, *gp130*, *hey1*, *myod*, and *gata2b* relative to *ef1a* from purified *fli1a*^+^ endothelial cells in 28 hpf Std and Notch1a morphants. Bars represent the means ± SEM of duplicate samples. **P < 0.01; ***p < 0.001; n.s., not significant. **h** WISH for *runx1* expression in the DA region in Std-, Tnfα-deficient embryos and Tnfα MO + *il6r* mRNA embryos at 28 hpf. **i** Qualitative phenotype distribution of embryos from **h** scored as in **d**

Notch signaling plays a crucial role in HSC specification^50–52^. Most recently, several proinflammatory factors have been implicated in HSC emergence, such as TNF-α, INF-γ, and TLR4, all of which are tightly associated with the Notch pathway^12–14^. In addition, one report has demonstrated that the Notch ligand Delta-1 regulates membrane-bound IL6R expression in myeloid progenitor cells in vitro^53^. These studies prompted us to investigate the relationship between Il6r and Notch in HSC development. We performed loss-of-function experiments with Il6r and Il6 in transgenic *tp1:eGFP* embryos to track cells with active Notching signaling^54^. We did not observe a reduced number of *tp1:eGFP*^*+*^*; kdrl:mCherry*^*+*^ HSCs in Il6- and Il6r-deficient embryos in the aortic floor at 32–36 hpf (Fig. 4a, b). The results indicate that Il6r is not upstream of Notch in HSC specification.

If Il6r lies downstream of Notch signaling, then the HSC defect through inhibiting Notch signaling should be rescued by the ectopic expression of *il6r*. We first treated embryos at 12 hpf using the Notch inhibitor DAPT to block Notch intracellular domain release from the plasma membrane, which led to the production of fewer HSCs in these embryos than in DMSO-treated siblings (Fig. 4c, d)^14,55^. In contrast, the enforced expression of *il6r* indeed rescued the depletion of *cmyb*^*+*^ HSCs along the DA region in DAPT-treated embryos at 48 hpf (Fig. 4c, d). A similar result was observed in *cd41:eGFP* transgenic embryos at 72 hpf (Fig. 4e, f), suggesting that Il6r may act downstream of Notch signaling in HSC development.

Notch1a promotes HSC specification in zebrafish embryos^13^. Notch1a-dependent *gata2b* is required to initiate *runx1* expression in the HE^56^. Thus, we attempted to explore potential mechanisms by which Notch1a induced *il6r* activation. We isolated *fli1a*^*+*^ ECs by FACS from 28 hpf transgenic *fli1a:eGFP* Notch1a-deficient embryos and performed qPCR for *il6r*, *gata2b*, and the known Notch target gene *hey1*. Intriguingly, all the transcripts were significantly lower in Notch1a morphants compared to controls (Fig. 4g). Additionally, Notch signaling is required downstream of Tnfα function during HSC emergence^13^. To investigate the functional relationship between Tnfα and Il6r, we enforced the expression of *il6r* in Tnfα-deficient embryos, and *runx1*^+^ cells in the aortic floor were restored at 28 hpf (Fig. 4h, i). Together, these results indicate that Notch1a indeed functions upstream of *il6r* by regulating its expression within the HE to specify HSC fate.

### HSC-independent neutrophils are the key source of Il6

**Fig. 5:**
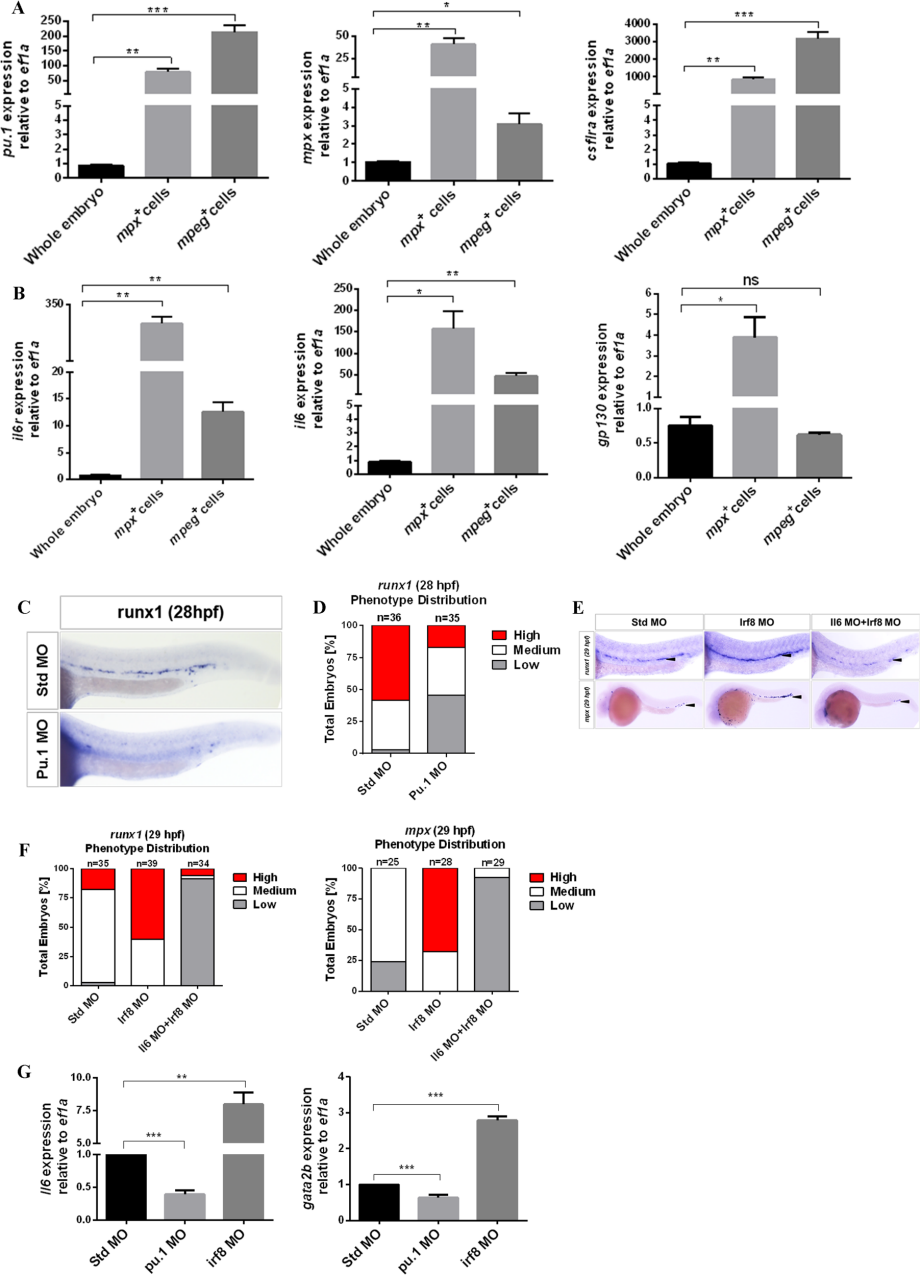
HSC-independent neutrophils play a key role in HSC specification. **a**, **b** qPCR for detecting the expression of genes, including *pu.1*, *csf1ra*, *mpx*, *il6r*, *il6*, and *gp130*, from purified HSC-independent macrophages (*mpeg1:GFP*^*+*^) and neutrophils (*mpx:GFP*^*+*^) at 30 hpf. Expression was normalized to *ef1a* and is presented relative to whole-embryo expression. Bars represent the means ± SEM of duplicate samples. *P < 0.05; **p < 0.01; ***p < 0.001; n.s., not significant. **c** Std and Pu.1 morphants were examined by WISH for *runx1* expression at 28 hpf. **d** Qualitative phenotype distribution of embryos from **c** scored with medium, high, and low *runx1* expression. Medium, white bar; high, red bar; low, gray bar. **e** Std, Irf8, and Il6 + Irf8 morphants were examined by WISH for *runx1* and *mpx* at 29 hpf. Black arrowheads denote *runx1*^*+*^ HSCs in the DA region and *mpx*^+^ neutrophils in the PBI and trunk regions. **f** Qualitative phenotype distribution of embryos from **e** scored as in **d**. **g** Whole-embryo expression of *il6* and *gata2b* relative to *ef1a* in Std, Pu.1, and Irf8 morphants at 28 hpf. Bars represent the means ± SEM of duplicate samples. **P < 0.01; ***p < 0.001

In adult organisms, IL6 is produced by almost all mature immune cells and HSPCs.^11^ While in the early development of zebrafish embryos, myeloid cells from primitive hematopoiesis and EMPs may be the key source of Il6 before HSC emergence. To investigate this hypothesis, we isolated *mpx:GFP* neutrophils and *mpeg1:GFP* MFs from embryos at 30 hpf by FACS and performed qPCR to detect the expression of *il6*, *il6r*, *gp130*, and myeloid-specific factors, including *pu.1*, *mpx*, and *csf1ra* (Fig. 5a, b). Compared with the cells of whole embryos, both *mpx*- and *mpeg1*-positive cells had higher expression of *il6* and *il6r*. This result suggests that HSC-independent myeloid cells produce a large amount of Il6.

To elucidate whether HSC-independent myeloid cells were the key source of Il6 needed for HSC emergence, we utilized a *pu.1* MO to ablate myeloid cells^38^, and then used the panleukocyte marker *l-plastin* by WISH to validate the efficiency of the MO (Fig. S3). Following the ablation of myeloid cells, the expression of *runx1* in the DA region was significantly lower in *pu.1* morphants compared to their control siblings (Fig. 5c, d). To further clarify which myeloid population was responsible for the reduction in HSC number, we utilized an *irf8* MO^13,37]^, which drives myeloid progenitor development almost entirely into neutrophils. Irf8 MO efficiency was validated using the neutrophil marker *mpx* at 29 hpf (Fig. 5e, f). Following the elevated number of neutrophils in the PBI and trunk regions, *runx1* within the DA region was significantly higher in Irf8-deficient embryos at 29 hpf compared to their control siblings. In agreement with our *il6* expression data, this result suggests that neutrophils may be the key source of Il6 needed for HSC emergence. As such, we examined *il6* expression in Pu.1- and Irf8 -deficient embryos. The expression of *il6* was decreased in Pu.1 morphants and increased in Irf8 morphants (Fig. 5g). Furthermore, simultaneously inhibiting the expression of *il6* and *irf8* resulted in a lack of *runx1* expression, with a decreased number of neutrophils in the same regions (Fig. 5e, f). These results indicate that *il6* derived from myeloid cells is necessary for HSC specification, and neutrophils are the key source of Il6. In addition, Il6 signaling is important for the development of HSC-independent neutrophils in the PBI and trunk regions.

## Discussion

Inflammatory factors play an important role in adult HSPC proliferation and differentiation into mature immune cells under stress-induced hematopoiesis^57,58^. Indeed, short-term HSCs and MPPs can produce multiple cytokines at a single-cell resolution^11^. However, for a long time, it remains unknown whether proinflammatory signaling could regulate embryonic HSC development. Most recently, several research groups have demonstrated that proinflammatory factors, such as TNF-α, IFN-γ, and TLR4, positively regulate HSC specification and emergence using zebrafish embryos^12–14^. As such, we also utilized the animal model to show that the depletion of Il6 and its cognate receptor Il6r leads to a failure of HSC emergence. These results support the hypothesis that HSCs evolved the capability of directly responding to proinflammatory factor signaling in embryonic development to defend against pathogenic infection after birth in almost all vertebrates.

Our study demonstrates that blocking Il6 signaling has a significant impact on HSC production, while two previous studies showed that *Il6*- and *Gp130*-mutant adult mice had slight defects of HSPCs in terms of proliferation and differentiation^23,24^. Genetic compensation can partly explain these phenomena. MOs for knocking down targeted genes in lower-vertebrate animal models can effectively avoid compensatory phenomena^59,60^. Therefore, we utilized the zebrafish embryo model to comprehensively explore the function of Il6 signaling in HSC emergence.

In our study, we identified that the HSC program can be rescued in Notch1-inhibited embryos by the ectopic expression of *il6r*, implying that *il6r* lies downstream of Notch in HSC specification. We demonstrated that Notch1a activates *il6r* by regulating its expression. In addition, we confirmed that Il6r acts downstream of Tnfα signaling. However, our study had some limitations. First, it remains to be determined whether Stat3/1 or other potential molecules might be downstream molecules of Il6r^14^. Second, to our knowledge, Il6 signaling can be divided into classic signaling and trans-signaling pathways^15^. Classic IL6R receptor signaling refers to the activation of cells mediated via the membrane-bound IL6R subunit. Trans-signaling denotes a process in which a soluble IL6R/IL6 complex binds to cells expressing only the GP130 subunit. Further studies will be required to determine which Il6 signaling participates in the process of HSC production.

With regard to the source of II6, one study showed that murine AGM-derived stromal cells could secrete multiple cytokines, including Il6^61^. Due to the lack of a recognized panel of specific protein markers, we failed to isolate these stromal cells. According to recent evidence, HSCs produce similar amounts of Il6 as vascular ECs or whole embryos^28,41^, so we deduced that HSC specification depends mainly on the paracrine effect of Il6. Indeed, we discovered for the first time that HSC-independent myeloid cells from primitive hematopoiesis and definitive EMPs are a major source of Il6. Neutrophils make a greater contribution to the source. Collectively, although sufficient evidence is available, it is necessary to precisely assess the contributions of different kinds of cells secreting Il6 using tissue-specific gene-knockout animal models.

In addition, we found an unexpected role for Il6 signaling in HSC-independent neutrophil development in the PBI and trunk regions. However, a similar function for Il6 signaling is not observed in primitive myeloid cells in the yolk sac. These results suggest that HSC-independent myeloid cells in different embryonic regions have different developmental orientations and functions in the adult immune system^62,63^. More studies are needed to elucidate the function of Il6 signaling in primitive and intermediate hematopoiesis.

In conclusion, our results suggest that Il6 signaling plays a pivotal role in HSC specification and emergence in zebrafish embryos. This information will be important for optimizing the strategy of HSC regeneration using the HE in vitro. However, it will still be a great challenge for the field to integrate various kinds of proinflammatory factor inputs in terms of the spatial and temporal requirements. More studies are necessary to optimize the protocol of HSC regeneration for HSC transplantation.

## Supplementary information

Supplementary figures

Supplementary tables

## Data Availability

All data generated or analyzed during this study are included in this published article and the supplementary information.
