# Supplementary material for: Interleukin-6 signaling regulates hematopoietic stem cell emergence
Source: Exp Mol Med. 2019 Oct 24;51(10):124. doi: 10.1038/s12276-019-0320-5 (PMC6813302; doi:10.1038/s12276-019-0320-5)
Supplement: Supplementary file 1 — Supplementary figures [file 12276_2019_320_MOESM1_ESM.docx]

SUPPLEMENTARY FIGURE LEGENDS

Supplementary Figure S1. Il6r and gp130 were enriched in in HEs, preHSCs and HSCs. We performed a secondary analysis using published data about single**-**cell RNA sequencing in an effort to investigate the expression levels il6, il6r and gp130 in hematopoietic endothelia (HEs), pre-hematopoietic stem cells (preHSCs, precursors of HSCs), and mature HSCs from mouse embryos at different developmental stages. Heat map analysis showed il6r and gp130 were higher in HEs, preHSCs and HSCs compared to vascular endothelial cells (ECs), whereas the expression of il6 was not detected because of the relatively low expression level in HEs and ECs. HE denotes hematopoietic endothelium, T1**-**preHSC denotes type 1 CD45- preHSC, T2-preHSC refers to type 2 CD45+ preHSC. E12_HSC refers to HSCs from mouse embryos at embryonic day 12 (E12), Adult HSC denote mature HSCs from adult mouse.

Supplementary Figure S2. Schematic representation of the predicted intron 2 insertion caused by the il6r splice MO, and DNA gel analysis showing the bigger il6r band following MO injection (n>30 embryos/condition).

Supplementary Figure S3. Validation of pu.1 MO effect. (A) Zebrafish zygotes injected with pu.1 and Std MO were subjected to WISH for *l-plastin* at 28 hpf. Black arrowheads denote leukocytes (*l-plastin*). (B) Qualitative phenotype distribution from (A). Medium, white bar; high, red bar; low, gray bar.

Figure S1


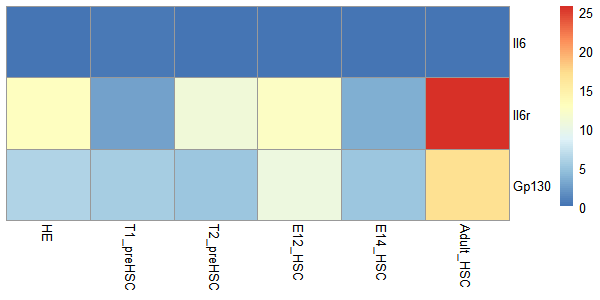


Figure S2


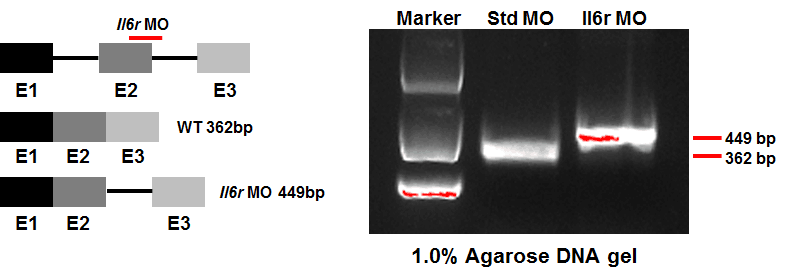


**
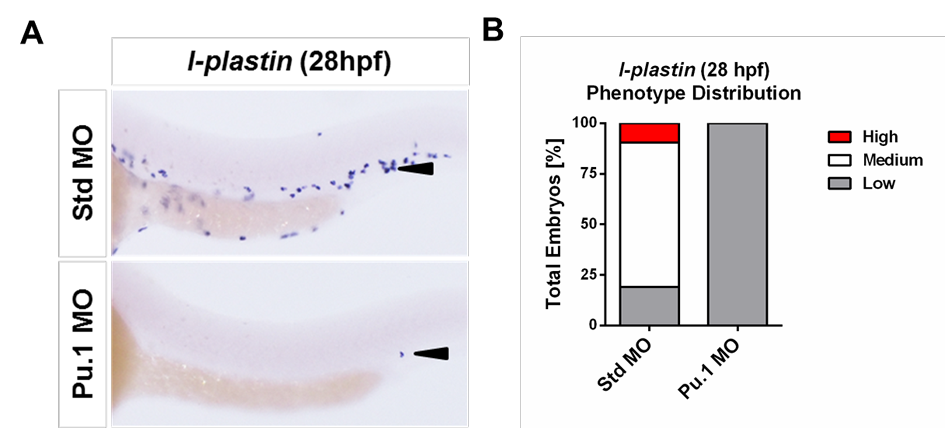
**Figure S3
