# Supplementary material for: Interleukin-6 signaling regulates hematopoietic stem cell emergence
Source: Exp Mol Med. 2019 Oct 24;51(10):124. doi: 10.1038/s12276-019-0320-5 (PMC6813302; doi:10.1038/s12276-019-0320-5)
Supplement: Supplementary file 2 — Supplementary tables [file 12276_2019_320_MOESM2_ESM.docx]

**Table S1. Zebrafish transgenic lines used in this study**

| **Official line name** | **Reference** |
| --- | --- |
| Tg(*cmyb:GFP*)^zf169^ | (North et al., 2007) |
| Tg(k*drl:HsHRAS-mCherry*)^s896^ | (Bertrand et al., 2010a) |
| Tg(*-6.0itga2b:eGFP*)^la2^ | (Lin et al., 2005) |
| Tg(*gata1:DsRed*)^sd2^ | (Traver et al., 2003) |
| Tg(*lck:eGFP*)^cz1^ | (Langenau et al., 2004) |
| Tg(*mpeg:eGFP*)^gl22^ | (Ellett et al., 2011) |
| Tg(*lyz:DsRed2*)^nz50^ | (Bo Yan et al.,2014) |
| Tg(*fli1a:eGFP*) | (Lawson et al., 2002) |
| Tg(*tp1-MmHbb:EGFP*)^um14^ | (Parsons et al., 2009) |

**Table S2. Zebrafish morpholino sequences**

| **Target gene** | **Morpholino sequence (5’-3’)** | **Reference** |
| --- | --- | --- |
| ***Il6r*** | **GTGTGCAAAAGTCCTTACCCCCTAC** | **This work** |
| ***Il6*** | **AGTATATAACAGCACCTGCTCGTCT** | **This work** |
| ***Irf8*** | **TTTGAAAATGGACGCATCCACCTC** | **Li et al., 2011** |
| ***Pu.1*** | **GATATACTGATACTCCATTGGTGGT** | **Jennifer et al., 2005** |
| ***Notch1a*** | **GTAGTGTTAAACTGTTACCTTGTGC** | **Ma and Jiang, 2007** |
| ***Tnfa*** | **GCAGGATTTTCACCTTATGGAGCGT** | **Lopez-Munoz et al., 2011** |

**Table S3. PCR primers for confirming the specificity of il6r MO**

| **Gene** | **Forward primer (Exon 2)** | **Reverse primer (Exon 3)** |
| --- | --- | --- |
| **Il6r** | **AGATGCTGGAAATTACAGTTGCT** | **TGTCATGTGGGTTAAAGTTGAGTA** |

**Table S4. PCR primers for amplifying the CDS of il6r into pCS2+ vector**

| **Gene** | **Forward primer** | **Reverse primer** |
| --- | --- | --- |
| ***Il6r*** | ggatcccatcgattc  GCCACCATGTGGACCCGATCTACACGC | atctcgagaggcctt  TTATTCATTTCTGTTTATTGTCAATGGC |

**Table S5. qPCR Primers in this study.**

| **Gene** | **Forward primer** | **Reverse primer** |
| --- | --- | --- |
| ***il6*** | **GACGTGGTATAAAGACAACTGGAAC** | **AAGGATAGGGAAGTGCTGGATG** |
| ***il6r*** | **TGGCTGTATGTGTCTTGTGC** | **TGGCTGAACAGGAAGGAAGT** |
| ***gp130*** | **TTGATGTGTGAAGCCTCCAG** | **TGGGTCTCTCAATCCTGGTT** |
| ***mpx*** | **TGATGTTTGGTTAGGAGGTG** | **GAGCTGTTTTCTGTTTGGTG** |
| ***csf1ra*** | **CATAACGACAGGTACGAGGCG** | **CGGGACAGGTGGTTCTGATT** |
| ***pu.1*** | **ATCACATCCCTCTAACCAATCC** | **GTCTCCATTTCGCAGAAGGTC** |
| ***gata2b*** | **ACCACCACACTCTGGAGAC** | **CTGTTGCGTGTCTGAATACC** |
| ***hey1*** | **GTTGAGCGGGACGGTGAT** | **GCTTAGATGCAGTAGGTGGAGTG** |
| ***myod*** | **GAGATTCTGAGAAACGCCAT** | **AAAATCCATCATGCCATCAGA** |
| ***ef1a*** | **GAGAAGTTCGAGAAGGAAGC** | **CGTAGTATTTGCTGGTCTCG** |

**Supplemental References**

1. North TE, Goessling W, Walkley CR, Lengerke C, Kopani KR, Lord AM，et al. Prostaglandin E2 regulates vertebrate haematopoietic stem cell homeostasis. *Nature* 2007; 447: 1007-1011.
2. [Bertrand JY](https://www.ncbi.nlm.nih.gov/pubmed/?term=Bertrand%20JY%5BAuthor%5D&cauthor=true&cauthor_uid=20154733), [Chi NC](https://www.ncbi.nlm.nih.gov/pubmed/?term=Chi%20NC%5BAuthor%5D&cauthor=true&cauthor_uid=20154733), [Santoso B](https://www.ncbi.nlm.nih.gov/pubmed/?term=Santoso%20B%5BAuthor%5D&cauthor=true&cauthor_uid=20154733), [Teng S](https://www.ncbi.nlm.nih.gov/pubmed/?term=Teng%20S%5BAuthor%5D&cauthor=true&cauthor_uid=20154733), [Stainier DY](https://www.ncbi.nlm.nih.gov/pubmed/?term=Stainier%20DY%5BAuthor%5D&cauthor=true&cauthor_uid=20154733), [Traver D](https://www.ncbi.nlm.nih.gov/pubmed/?term=Traver%20D%5BAuthor%5D&cauthor=true&cauthor_uid=20154733). Haematopoietic stem cells derive directly from aortic endothelium during development. *Nature* 2010; 464: 108-111.
3. [Lin HF](https://www.ncbi.nlm.nih.gov/pubmed/?term=Lin%20HF%5BAuthor%5D&cauthor=true&cauthor_uid=16099879), [Traver D](https://www.ncbi.nlm.nih.gov/pubmed/?term=Traver%20D%5BAuthor%5D&cauthor=true&cauthor_uid=16099879), [Zhu H](https://www.ncbi.nlm.nih.gov/pubmed/?term=Zhu%20H%5BAuthor%5D&cauthor=true&cauthor_uid=16099879), [Dooley K](https://www.ncbi.nlm.nih.gov/pubmed/?term=Dooley%20K%5BAuthor%5D&cauthor=true&cauthor_uid=16099879), [Paw BH](https://www.ncbi.nlm.nih.gov/pubmed/?term=Paw%20BH%5BAuthor%5D&cauthor=true&cauthor_uid=16099879), [Zon LI](https://www.ncbi.nlm.nih.gov/pubmed/?term=Zon%20LI%5BAuthor%5D&cauthor=true&cauthor_uid=16099879), et al. Analysis of thrombocyte development in CD41-GFP transgenic zebrafish. *Blood* 2005; 106: 3803-3810.
4. [Traver D](https://www.ncbi.nlm.nih.gov/pubmed/?term=Traver%20D%5BAuthor%5D&cauthor=true&cauthor_uid=14608381), [Paw BH](https://www.ncbi.nlm.nih.gov/pubmed/?term=Paw%20BH%5BAuthor%5D&cauthor=true&cauthor_uid=14608381), [Poss KD](https://www.ncbi.nlm.nih.gov/pubmed/?term=Poss%20KD%5BAuthor%5D&cauthor=true&cauthor_uid=14608381), [Penberthy WT](https://www.ncbi.nlm.nih.gov/pubmed/?term=Penberthy%20WT%5BAuthor%5D&cauthor=true&cauthor_uid=14608381), [Lin S](https://www.ncbi.nlm.nih.gov/pubmed/?term=Lin%20S%5BAuthor%5D&cauthor=true&cauthor_uid=14608381), [Zon LI](https://www.ncbi.nlm.nih.gov/pubmed/?term=Zon%20LI%5BAuthor%5D&cauthor=true&cauthor_uid=14608381). Transplantation and in vivo imaging of multilineage engraftment in zebrafish bloodless mutants. *Nature Immunology* 2003;4: 1238-1246.
5. [Langenau DM](https://www.ncbi.nlm.nih.gov/pubmed/?term=Langenau%20DM%5BAuthor%5D&cauthor=true&cauthor_uid=15123839), [Ferrando AA](https://www.ncbi.nlm.nih.gov/pubmed/?term=Ferrando%20AA%5BAuthor%5D&cauthor=true&cauthor_uid=15123839), [Traver D](https://www.ncbi.nlm.nih.gov/pubmed/?term=Traver%20D%5BAuthor%5D&cauthor=true&cauthor_uid=15123839), [Kutok JL](https://www.ncbi.nlm.nih.gov/pubmed/?term=Kutok%20JL%5BAuthor%5D&cauthor=true&cauthor_uid=15123839), [Hezel JP](https://www.ncbi.nlm.nih.gov/pubmed/?term=Hezel%20JP%5BAuthor%5D&cauthor=true&cauthor_uid=15123839), [Kanki JP](https://www.ncbi.nlm.nih.gov/pubmed/?term=Kanki%20JP%5BAuthor%5D&cauthor=true&cauthor_uid=15123839)，et al. In vivo tracking of T cell development, ablation, and engraftment in transgenic zebrafish. *Proc. Natl. Acad. Sci. USA* 2004; 101:7369-7374.
6. [Ellett F](https://www.ncbi.nlm.nih.gov/pubmed/?term=Ellett%20F%5BAuthor%5D&cauthor=true&cauthor_uid=21084707), [Pase L](https://www.ncbi.nlm.nih.gov/pubmed/?term=Pase%20L%5BAuthor%5D&cauthor=true&cauthor_uid=21084707), [Hayman JW](https://www.ncbi.nlm.nih.gov/pubmed/?term=Hayman%20JW%5BAuthor%5D&cauthor=true&cauthor_uid=21084707), [Andrianopoulos A](https://www.ncbi.nlm.nih.gov/pubmed/?term=Andrianopoulos%20A%5BAuthor%5D&cauthor=true&cauthor_uid=21084707), [Lieschke GJ](https://www.ncbi.nlm.nih.gov/pubmed/?term=Lieschke%20GJ%5BAuthor%5D&cauthor=true&cauthor_uid=21084707). mpeg1 promoter transgenes direct macrophage-lineage expression in zebrafish. *Blood* 2011; 117: 49-56.
7. Yan B, Han P, Pan L, [Lu W](https://www.ncbi.nlm.nih.gov/pubmed/?term=Lu%20W%5BAuthor%5D&cauthor=true&cauthor_uid=24835391), [Xiong J](https://www.ncbi.nlm.nih.gov/pubmed/?term=Xiong%20J%5BAuthor%5D&cauthor=true&cauthor_uid=24835391), [Zhang M](https://www.ncbi.nlm.nih.gov/pubmed/?term=Zhang%20M%5BAuthor%5D&cauthor=true&cauthor_uid=24835391), et al. IL-1β and reactive oxygen species differentially regulate neutrophil directional migration and Basal random motility in a zebrafish injury-induced inflammation model. *Journal of Immunology* 2014; 192: 5998-6008.
8. Lawson N D, Weinstein B M. In vivo imaging of embryonic vascular development using transgenic zebrafish. *Developmental Biology* 2002; 248:307-318.
9. [Parsons MJ](https://www.ncbi.nlm.nih.gov/pubmed/?term=Parsons%20MJ%5BAuthor%5D&cauthor=true&cauthor_uid=19595765), [Pisharath H](https://www.ncbi.nlm.nih.gov/pubmed/?term=Pisharath%20H%5BAuthor%5D&cauthor=true&cauthor_uid=19595765), [Yusuff S](https://www.ncbi.nlm.nih.gov/pubmed/?term=Yusuff%20S%5BAuthor%5D&cauthor=true&cauthor_uid=19595765), [Moore JC](https://www.ncbi.nlm.nih.gov/pubmed/?term=Moore%20JC%5BAuthor%5D&cauthor=true&cauthor_uid=19595765), [Siekmann AF](https://www.ncbi.nlm.nih.gov/pubmed/?term=Siekmann%20AF%5BAuthor%5D&cauthor=true&cauthor_uid=19595765), [Lawson N](https://www.ncbi.nlm.nih.gov/pubmed/?term=Lawson%20N%5BAuthor%5D&cauthor=true&cauthor_uid=19595765), et al. Notch-responsive cells initiate the secondary transition in larval zebrafish pancreas. *Mechanisms of Development* 2009; 126: 898-912.
10. [López-Muñoz A](https://www.ncbi.nlm.nih.gov/pubmed/?term=L%C3%B3pez-Mu%C3%B1oz%20A%5BAuthor%5D&cauthor=true&cauthor_uid=21354627), [Sepulcre MP](https://www.ncbi.nlm.nih.gov/pubmed/?term=Sepulcre%20MP%5BAuthor%5D&cauthor=true&cauthor_uid=21354627), [Roca FJ](https://www.ncbi.nlm.nih.gov/pubmed/?term=Roca%20FJ%5BAuthor%5D&cauthor=true&cauthor_uid=21354627), [Figueras A](https://www.ncbi.nlm.nih.gov/pubmed/?term=Figueras%20A%5BAuthor%5D&cauthor=true&cauthor_uid=21354627), [Meseguer J](https://www.ncbi.nlm.nih.gov/pubmed/?term=Meseguer%20J%5BAuthor%5D&cauthor=true&cauthor_uid=21354627), [Mulero V](https://www.ncbi.nlm.nih.gov/pubmed/?term=Mulero%20V%5BAuthor%5D&cauthor=true&cauthor_uid=21354627). Evolutionary conserved pro-inflammatory and antigen presentation functions of zebrafish IFNγ revealed by transcriptomic and functional analysis. [[*Molecular Immunology*](https://www.sciencedirect.com/science/journal/01615890).](https://www.ncbi.nlm.nih.gov/pubmed/?term=Lopez-Munoz+2011+tnfa) 2011; 48:1073-83.
